# Impact of Overweight and Obesity on Disease Outcome in the Pediatric Swiss Inflammatory Bowel Disease Cohort

**DOI:** 10.1097/PG9.0000000000000193

**Published:** 2022-03-31

**Authors:** Thea von Graffenried, Alain M. Schoepfer, Jean-Benoit Rossel, Thomas Greuter, Ekaterina Safroneeva, Sébastien Godat, Sarah Henchoz, Stephan R. Vavricka, Christiane Sokollik, Johannes Spalinger, Christian P. Braegger, Andreas Nydegger

**Affiliations:** From the * Division of Pediatric Gastroenterology Hepatology and Nutrition, Centre Hospitalier Universitaire Vaudois [CHUV] and University of Lausanne, Lausanne, Switzerland; † Division of Gastroenterology and Hepatology, Centre Hospitalier Universitaire Vaudois [CHUV] and University of Lausanne, Lausanne, Switzerland; ‡ Clinical Trials Unit, Institute of Social and Preventive Medicine, University of Bern, Bern, Switzerland; § Division of Gastroenterology and Hepatology, University Hospital Zurich, Zurich, Switzerland; ∥ Institute of Social and Preventive Medicine, University of Bern, Bern, Switzerland; ¶ Center for Gastroenterology and Hepatology, Zurich, Switzerland; # Division of Pediatric Gastroenterology, Hepatology and Nutrition, University Children’s Hospital, Inselspital, University of Bern, Bern, Switzerland; ** Division of Pediatric Gastroenterology, Hepatology and Nutrition, Children’s Hospital LUKS, Lucerne, Switzerland; †† Nutrition Research Unit, University Children’s Hospital Zurich, Zurich, Switzerland.

**Keywords:** Crohn’s disease, ulcerative colitis, body mass index, disease activity, quality of life

## Abstract

Given the paucity of data, we aimed to assess the impact of obesity on disease activity, complications, and quality of life (QoL) in pediatric inflammatory bowel disease (IBD) patients.

**Methods:**

Prospective analysis of pediatric IBD patients. Patients were categorized into 4 groups according to the World Health Organization (WHO) child growth standards: obese, overweight, normal weight, and underweight.

**Results:**

Three hundred twenty-seven pediatric patients were included (146 with Crohn’s disease [CD], 181 with ulcerative colitis of whom 13 [4%] were underweight, 272 [83.2%] had normal weight, 22 [6.7%] were overweight, and 20 [6.1%] were obese). Compared with normal weight patients, obese ulcerative colitis had a significantly higher clinical but not biological disease activity nor severity. Compared with normal weight patients, overweight/obese CD patients did not have higher clinical or biological disease activity nor severity. Perianal abscesses and surgery for this purpose were more frequently observed in overweight/obese CD patients compared with normal weight controls. Overweight/obese IBD patients were similarly hospitalized in the last 12 months compared with normal weight controls.

**Conclusions:**

Prevalence of overweight/obesity was 12.8% in pediatric IBD patients. Obesity was not associated with a decrease in disease remission rates nor an increase in the risk of complicated disease progression in IBD pediatric patients, except for the occurrence of perianal abscesses and related surgery in CD patients.

**What Is Known**

- Prevalence of overweight and obesity in children has increased dramatically in recent decades, including in the inflammatory bowel disease (IBD) population.
- Data on the role and impact of obesity in pediatric IBD population are conflicting.

**What Is New**

- Significant association was found between clinical activity and obesity in ulcerative colitis (UC) patients with, however, low PUCAI score values corresponding to clinical remission in both obese and normal weight patients. This association was not found in Crohn’s disease (CD) patients.
- Overweight and obese CD patients suffered more frequently from perianal abscesses and related surgery compared with normal weight patients.
- Among CD and UC, disease severity was comparable in overweight/obese and normal weight patients.
- Pediatric CD and UC patients with overweight/obesity underwent similarly IBD-related hospitalization in the last 12 months compared with normal weight patients.

## INTRODUCTION

Worldwide, the prevalence of overweight and obesity in children has increased dramatically during the last decades. For the general population of the United States, the prevalence of obesity increased from 8% to 14% in 6- to 12-year-old children between 1976 and 1994 and even reached 18.4% in 2015–2016 (1–3). In comparison, a Swiss national study revealed a prevalence of overweight and obesity of 15.9% and 5.3%, respectively, between 2017 and 2018 in children of the same age (3). In parallel with the obesity epidemic, a similar rising trend has been observed in the incidence and prevalence of inflammatory bowel disease (IBD). In a multicenter cohort of children with IBD of the United States, the prevalence of overweight and obesity was 23.6% (4). By contrast, another multicenter retrospective study conducted in Poland estimated the prevalence of overweight and obesity in new-onset pediatric IBD at 8.4% (5). Crohn’s disease (CD) and ulcerative colitis (UC) are immune-mediated diseases categorized by chronic inflammation and progressive damage to the gastrointestinal tract, which can contribute to weight loss and malnutrition (4,6). Given the increasing prevalence of obesity worldwide, its association with IBD is becoming more important. Recent studies suggest that changes in the intestinal immune system and suspected dysbiosis in the pathophysiology of IBD may independently contribute to obesity (7,8). Similarly to the general population, obesity is emerging as a serious problem among individuals with immune-mediated diseases, such as IBD, but its impact remains limited and inconclusive. Existing data on the role and impact of obesity in adult IBD population are conflicting with studies suggesting increased disease flares and complications, whereas other studies show no impact or even reduced need of surgery in obese IBD patients (9–11). Literature on impact of obesity in pediatric IBD is even more limited. Given the scarce evidence on this topic, we aimed to determine the prevalence of overweight and obesity in our large pediatric IBD population and assess the impact of overweight and obesity on disease phenotypes and clinical outcomes.

## METHODS

### Study Design

Swiss Inflammatory Bowel Disease Cohort Study (SIBDCS) has been including IBD patients from all regions of Switzerland with pediatric enrollment starting in 2006. SIBDCS is a national prospective cohort study on IBD patients, and provides up-to-date information regarding different aspects of IBD in Switzerland for the Swiss and international scientific community, public health authorities, and medical staff (12). Inclusion criteria are detailed in the recently published cohort profile update (13). Ethics approval was obtained for the study protocol by the ethics committee of the cantons or regions in which patients were included (14).

### Study Population and Outcomes Assessed

Collected data were recorded using a Microsoft Access database [Access 2000, Microsoft Switzerland, Wallisellen, Switzerland] at the data center of SIBDCS and Swiss Paediatric IBD Cohort Study (SPIBDCS), which is located at the Institute of Social and Preventive Medicine at the University of Lausanne. For this article, the analysis was based on the data obtained from pediatric IBD patients enrolled into SPIBDCS between May 2006 and May 2020 or until the age of 18 years.

Patients included in SPIBDCS undergo structured assessment of disease activity at enrollment and during annual follow-up visits. Detailed questionnaires are completed by the patient, the caregivers, and the respective physician. For the present analysis, we included underweight, normal weight, overweight and obese IBD pediatric patients. As this study focuses on overweight and obese pediatric patients, underweight patients were only analyzed demographically. The following inclusion criteria were applied: (1) diagnosis of CD or UC; (2) enrollment into SPIBDCS between 2006 and 2020; (3) available data on weight, height, body mass index (BMI), gender and age at enrollment and during follow up. For the longitudinal analyses of complications development, we excluded patients with complications at baseline.

Using BMI at enrollment and at latest follow up, we categorized patients into 4 groups according to the World Health Organization (WHO) child growth standards: obese, overweight, normal weight, and underweight. Obesity was defined as BMI >97th percentile, overweight as 90th to 97th percentile, normal weight as 3th to 90th percentile, and underweight as <3th percentile (15).

The following data were recorded for all eligible pediatric patients: demographic variables (gender, age at enrollment), disease location at diagnosis according to the Paris classification (L1-L4 in CD and E1-E3 in UC) (16), disease behavior in CD (B1–B3), disease duration (determined from time of diagnosis), extraintestinal manifestations (EIM) (arthritis, uveitis/iritis, pyoderma gangrenosum, erythema nodosum, oral ulcers, ankylosing spondylitis, primary sclerosing cholangitis), IBD drug history (current and past therapies), disease severity (antitumor necrosis factor [TNF] treatment, IBD-related surgery). Clinical disease activity was measured by the Pediatric Crohn’s Disease Activity Index for CD and Pediatric Ulcerative Colitis Activity Index (PUCAI) for UC (14,17). Biological disease activity was measured by C-reactive protein and fecal calprotectin. The quality of life (QoL) assessment was based on KIDSCREEN-GROUP 2004 and IMPACT III questionnaires, which are two validated and reliable instruments commonly used among pediatric IBD patients (18,19). Other assessments considering the psychologic state of the patient, coping strategies, and stress symptoms by Depressionsinventar für Kinder und Jugendliche, KIDCOPE, The University of California at Los Angeles Post-traumatic Stress Disorder Reaction Index scores, respectively, were also used (20–22). To assess whether or not obesity affects disease course, complicated disease course was defined as development of any of the following outcomes in CD patients: fistulizing phenotype (perianal fistula and nonperianal fistula), stricturing phenotype (intestinal stenosis), intestinal surgery (fistula and abscess surgery), and CD-related hospitalizations over the last 12 months. In UC patients, complicated disease course was defined as either undergoing colectomy or UC-related hospitalizations over the last 12 months. Survival analysis, represented by the occurrence of EIM, surgery, or fistula, and stratified by diagnosis, were performed.

### Statistical Analysis

Data were retrieved from the database of the SIBDCS at the Institute of Social and Preventive Medicine at the University of Lausanne, Switzerland. All statistical analyses were performed by the cohort statistician using the statistical program Stata (version 16.1, College Station, Texas). Quantitative data distribution was analyzed using Normal-QQ-Plots. Results of quantitative data are presented as median, interquartile ranges (IQR), minimum, and maximum values. Differences in distribution between two groups were evaluated using the Wilcoxon/Mann-Whitney rank test. Moreover, Kruskal-Wallis test was used in case of distribution comparisons between more than two groups. For categorical outcomes, differences in observed frequencies between groups were compared using the Fisher’s exact test, due to the small number of observations in some groups (n < 5). Categorical data are summarized as percentage of the total group. To assess development of complications, Kaplan-Meier curves and Cox proportional hazard models were computed (data not shown). The log-rank test was used to detect overall statistical difference in estimates. Bonferroni method was applied in order to avoid random significance generation where applicable. For the purpose of this study, a *P* value <0.05 was considered statistically significant. In Table 2, the four groups (underweight, normal, overweight, obese) all together were compared. For each characteristic, the null hypothesis is that the four groups have the same distribution. A significant *P* value result for a characteristic means that at least one group is different from the other groups, according to this characteristic. Hazard ratios (95% CI; *P* value) of the BMI decile in time-to-event were analyzed. We focus on the occurrence of arthritis, oral ulcers, any EIM, and any surgery. We also focus on the occurrence of any fistula in CD patients. Crude hazard ratios as well as adjusted hazard ratios were calculated. Adjustment was done for age, sex, steroid intake, and initial disease location.

## RESULTS

### Baseline Characteristics

Distribution of BMI categories at baseline and at latest follow up are presented in Table 1. None of the underweight patients at enrollment became overweight or obese at latest follow up and conversely, none of the overweight/obese patients at enrollment became underweight at latest follow up. Between enrollment and latest follow up 50% of overweight patients normalized their BMI value and 50% of obese patients did not change their BMI category. Demographic and disease characteristics are shown in Table 2 for the entire IBD population and stratified according to IBD subtype (Supplemental Digital Tables 1 and 2, http://links.lww.com/PG9/A79). A total of 327 pediatric patients were included (median BMI of 17.6 kg/m^2^, IQR, 16.0–20.4, range 12.3–33.5), of whom 13 (4%) were underweight (median BMI of 14.2 kg/m^2^, IQR 13.1–15.4, range 12.3–15.6), 272 (83.2%) presented with normal weight (median BMI of 17.5 kg/m^2^, IQR 16.0–19.5, range 13.2–24.7), 22 (6.7%) were overweight (median BMI of 22.4 kg/m^2^, IQR 19.8–24.2, range 17.1–26.8), and 20 (6.1%) were obese (median BMI of 23.6 kg/m^2^, IQR 21.3–27.1, range 18.1–33.5). Out of the 42 overweight and obese patients, 17 (40.5%) had CD and 25 (59.5%) had UC. Among all IBD patients, more male patients were observed when compared with normal weight individuals (80% versus 49.3%, *P* = 0.041). Median age at diagnosis of all IBD patients was 9.5 (IQR 5.3–12.2) and 10.1 (IQR 5.2–11.2) years with disease duration of 1.6 (IQR 0.6–2.8) and 0.7 (IQR 0.6–2) years in overweight and obese patients, respectively.

**TABLE 1.** Distribution of BMI categories at baseline (rows) and at latest follow up (columns)

	Underweight at latest follow up	Normal at latest follow up	Overweight at latest follow up	Obese at latest follow up	Total
Underweight at baseline	5 (38.5%)	8 (61.5%)	0 (0%)	0 (0%)	13
Normal at baseline	7 (2.6%)	250 (91.9%)	11 (4.0%)	4 (1.5%)	272
Overweight at baseline	0 (0%)	11 (50.0%)	9 (40.9%)	2 (9.1%)	22
Obese at baseline	0 (0%)	5 (25.0%)	5 (25.0%)	10 (50.0%)	20
Total	12 (3.7%)	274 (83.8%)	25 (7.6%)	16 (4.9%)	327

BMI = body mass index.

Median follow up of IBD patients was 2.0 years (IQR 0.9–3.9, range 0–9.7) with a median follow-up time of 2.9 years (IQR 1.7–4.1, range 0–8.3) in obese patients and a median follow-up time of 2.5 years (IQR 1.4–3.4, range 0–9.7) in overweight patients. Obese patients, and more specifically obese CD patients, were more likely to be treated with steroids than underweight patients (80% versus 38.5%, *P* = 0.017; 85.7% versus 22.2%, *P* = 0.021).

### Disease Severity

There was no significant difference between overweight/obese patients and normal weight patients who received anti-TNF drugs. IBD-related surgery was more frequently performed in underweight and overweight patients when compared with normal weight and obese patients (*P* = 0.048, Table 2). Among CD patients, the majority of surgery was performed in overweight patients, whereas in UC patients, no IBD-related surgery was reported in obese patients.

**TABLE 2.** Baseline characteristics of all IBD patients

BMI category at enrollment	Underweight	Normal	Overweight	Obese	*P*
Number of patients, n (%)	13 (4.0)	272 (83.2)	22 (6.7)	20 (6.1)	
Gender, n (%)					
Male	8 (61.5)	134 (49.3)	13 (59.1)	16 (80.0)	0.041
Female	5 (38.5)	138 (50.7)	9 (40.9)	4 (20.0)	
Diagnosis (at latest follow up), n (%)					
CD	9 (69.2)	120 (44.1)	10 (45.5)	7 (35.0)	0.271
UC	4 (30.8)	152 (55.9)	12 (54.5)	13 (65.0)	
Age at diagnosis (y), median, IQR (range)	12.1, 6.0–13.8, 2.5–15.4	11.4, 8.3–13.5, 0.5–16.3	9.5, 5.3–12.2, 3.1–17.2	10.1, 5.2–11.2, 3.2–14.9	0.034
Age (y) at enrollment, median, IQR (range)	12.9, 7.6–14.5, 3.3–16.5	12.8, 10.0–14.7, 1.3–17.1	12.8, 9.5–13.6, 3.9–17.6	11.1, 8.4–12.5, 3.8–15.3	0.072
Disease duration (y) at enrollment, median, IQR (range)	1.1, 0.7–1.6, 0.3–4.9	0.8, 0.5–1.7, 0.2–9.9	1.6, 0.6–2.8, 0.4–9.7	0.7, 0.6–2.0, 0.3–5.3	0.244
Follow-up time (y), median, IQR (range)	2.0, 1.9–4.2, 0–5.5	1.9, 0.8–3.9, 0–9.5	2.5, 1.4–3.4, 0–9.7	2.9, 1.7–4.1, 0–8.3	0.369
Current treatments, n (%)					
5-ASA oral	2 (15.4)	106 (39.0)	9 (40.9)	9 (45.0)	0.338
5-ASA topical	1 (7.7)	24 (8.8)	4 (18.2)	2 (10.0)	0.462
Prednisone	1 (7.7)	46 (16.9)	4 (18.2)	2 (10.0)	0.812
Budesonide	0 (0)	3 (1.1)	0 (0)	1 (5.0)	0.367
Thiopurines	4 (30.8)	91 (33.5)	8 (36.4)	8 (40.0)	0.927
Methotrexate	0 (0)	35 (12.9)	2 (9.1)	1 (5.0)	0.551
CNI	0 (0)	1 (0.4)	0 (0)	0 (0)	1.000
Anti-TNF	6 (46.2)	109 (40.1)	7 (31.9)	7 (35.0)	0.814
Vedolizumab	0 (0)	7 (2.6)	1 (4.5)	2 (10.0)	0.180
Ustekinumab	0 (0)	3 (1.1)	1 (4.5)	0 (0)	0.523
Ever treated with…, n (%)					
5-ASA oral	9 (69.2)	170 (62.5)	16 (72.7)	15 (75.0)	0.592
5-ASA topical	2 (15.4)	80 (29.4)	9 (40.9)	7 (35.0)	0.419
Prednisone	5 (38.5)	191 (70.2)	11 (50.0)	16 (80.0)	0.017
Budesonide	2 (15.4)	42 (15.4)	4 (18.2)	6 (30.0)	0.377
Thiopurines	11 (84.6)	188 (69.1)	13 (59.1)	16 (80.0)	0.342
Methotrexate	3 (23.1)	59 (21.7)	6 (27.3)	2 (10.0)	0.556
CNI	1 (7.7)	9 (3.3)	0 (0)	0 (0)	0.512
Anti-TNF	7 (53.8)	132 (48.5)	10 (45.5)	8 (40.0)	0.848
Vedolizumab	1 (7.7)	10 (3.7)	2 (9.1)	2 (10.0)	0.130
Ustekinumab	0 (0)	3 (1.1)	1 (4.5)	0 (0)	0.523
History of IBD-related surgery, n (%)					
No	10 (76.9)	249 (91.5)	17 (77.3)	18 (90.0)	0.048
Yes	3 (23.1)	23 (8.5)	5 (22.7)	2 (10.0)	
Smoking status at latest follow up, n (%)					
Nonsmoker	12 (92.3)	252 (92.7)	21 (95.5)	20 (100)	0.612
Smoker	1 (7.7)	15 (5.5)	0 (0)	0 (0)	
Unknown	0 (0)	5 (1.8)	1 (4.5)	0 (0)	

5-ASA = aminosalicylates; BMI = body mass index; CD = Crohn’s disease; CNI = calcineurin inhibitor; IBD = inflammatory bowel disease; IQR = interquartile range; TNF = tumor necrosis factor; UC = ulcerative colitis.

### Disease Behavior, Disease Activity, and EIM

Disease behavior and disease location were comparable between overweight/obese and normal weight CD patients (Table 3). No difference was found with respect to clinical disease activity (measured by Pediatric Crohn’s Disease Activity Index) as well as biological activity values (measured by C-reactive protein and fecal calprotectin). EIM were found with comparable frequencies in both groups except for arthritis with higher prevalence in obese CD compared with normal BMI patients (47.1% versus 21.7%, *P* = 0.034).

**TABLE 3.** Disease activity and behavior in CD patients

BMI category at enrollment	Normal BMI (n = 120)	Overweight/obese (n = 17)	*P*
Fistulas (ever), n (%)			
Perianal	16 (13.3)	3 (17.6)	0.706
Any	18 (15.0)	4 (23.5)	0.477
Perianal abscess (ever), n (%)			
No	113 (94.2)	10 (58.8)	<0.001
Yes	7 (5.8)	7 (41.2)	
Intestinal surgery (ever), n (%)			
No	118 (98.3)	16 (94.1)	0.330
Yes	2 (1.7)	1 (5.9)	
Surgery for fistula or abscess (ever), n (%)			
No	115 (95.8)	11 (64.7)	<0.001
Yes	5 (4.2)	6 (35.3)	
Bowel stenosis (ever), n (%)			
No	113 (94.2)	15 (88.2)	0.309
Yes	7 (5.8)	2 (11.8)	
Behavior (latest follow up), n (%)			
B1 inflammatory	105 (87.5)	16 (94.1)	0.148
B1p	12 (10.0)	0 (0)	
B2 stricturing	2 (1.7)	0 (0)	
B2p	0 (0)	0 (0)	
B3 penetrating	1 (0.8)	0 (0)	
B3p	0 (0)	1 (5.9)	
Disease location at diagnosis, n (%)			
L1 (ileal)	14 (12.1)	1 (6.3)	0.692
L2 (colonic)	15 (12.9)	2 (12.5)	
L3 (ileo-colonic)	84 (72.4)	12 (75.0)	
L4 (upper GI only)	3 (2.6)	1 (6.3)	
Unknown/unclear	4	1	
PCDAI score (latest follow up), median, IQR (range)	2.5, 0–5, 0–40	2.5, 0–5, 0–10	0.983
Number of soft stools per day, median, IQR (range) (latest follow up)	0, 0–5, 0–60	0, 0–9, 0–30	0.896
CRP in mg/L, median, IQR (range) (latest follow up)	3, 2–5, 0.3–71	3.1, 3–6, 0.4–37	0.561
Fecal calprotectin in µg/g, median, IQR (range) (latest follow up)	147, 42–767, 11–4939	385, 78–821, 17–1192	0.792
Extraintestinal manifestations (ever), n (%)			
Arthritis	26 (21.7)	8 (47.1)	0.034
Uveitis/iritis	5 (4.2)	0 (0)	1.000
Pyoderma gangrenosum	0 (0)	0 (0)	–
Erythema nododum	5 (4.2)	0 (0)	1.000
Oral ulcers	31 (25.8)	6 (35.3)	0.397
Axial spondylarthropathy	3 (2.5)	0 (0)	1.000
Primary sclerosing cholangitis	1 (0.8)	0 (0)	1.000
Any	53 (44.2)	9 (52.9)	0.605
Medical resource consumption over the last 12 mo, n (%)			
Hospitalization	13 (10.8)	2 (11.8)	1.000
No hospitalization	107 (89.2)	15 (88.2)	

BMI = body mass index; CD = Crohn’s disease; CRP = C-reactive protein; GI = gastrointestinal; IQR = interquartile range; PCDAI = Pediatric Crohn’s Disease Activity Index.

We observed a difference in clinical disease activity (measured by PUCAI) between obese and normal weight UC patients (*P* = 0.034), with median values corresponding to clinical remission in the different groups. However, there was no significant difference in terms of biological activity score, number of bloody stools, and presence of any EIMs. In analogy to the findings in CD patients, disease location was not different when comparing normal weight UC patients with overweight/obese UC patients (Table 4).

**TABLE 4.** Disease activity in UC patients

BMI category at enrollment	Normal (n = 152)	Overweight/obese (n = 25)	*P*
Intestinal surgery (ever), n (%)			
No	149 (98.0)	25 (100)	1.000
Yes	3 (2.0)	0 (0)	
Disease location at diagnosis, n (%)			
Proctitis	13 (9.2)	1 (4.6)	0.868
Left-sided	29 (20.6)	5 (22.7)	
Pancolitis	99 (70.2)	16 (72.7)	
Unknown/Unclear	11	3	
PUCAI score (latest follow up), median, IQR (range)	0, 0–5, 0–55	5, 0–15, 0–55	0.034
Number of soft stools per day (latest follow up), n (%)			
1–2	70 (88.6)	10 (90.9)	0.748
3–4	5 (6.3)	1 (9.1)	
5–6	3 (2.8)	0 (0)	
7–9	1 (1.3)	0 (0)	
Missing	73	14	
CRP in mg/L (latest follow up), median, IQR (range)	3, 2–5, 0–53	4, 2.8–7, 0.3–110	0.106
Fecal calprotectin in µg/g (latest follow up), median, IQR (range)	146, 39–1010, 0.8–10,672	239, 56–605, 5–2095	0.726
Extraintestinal manifestations (ever), n (%)			
Arthritis	14 (9.2)	3 (12.0)	0.713
Uveitis/Iritis	2 (1.3)	0 (0)	1.000
Pyoderma gangrenosum	1 (0.7)	0 (0)	1.000
Erythema nodosum	0 (0)	0 (0)	–
Oral ulcers	17 (11.2)	1 (4.0)	0.475
Axial spondylarthropathy	0 (0)	0 (0)	–
Primary sclerosing cholangitis	10 (6.6)	0 (0)	0.361
Any	36 (23.7)	3 (12.0)	0.297
Medical resource consumption over the last 12 months, n (%)			
Hospitalization	16 (10.6)	4 (16.0)	0.493
No hospitalization	135 (89.4)	21 (84.0)	

BMI = body mass index; CRP = C-reactive protein; IQR = interquartile range; PUCAI = Pediatric Ulcerative Colitis Activity Index; UC = ulcerative colitis.

### Disease Outcome, Complications and Hospitalization Rates

In CD, overweight/obesity was significantly associated with development of perianal abscesses (*P* < 0.001, Table 3). Similarly, overweight/obese patients with abscesses have more surgeries related to this condition. Of the 14 patients with abscesses, 11 have surgery for this purpose (*P* < 0.001, Table 3). Development of stricturing phenotype as a complication of the disease was similar between the two groups. We observed a similar rate of hospitalizations in the past 12 months in both, overweight/obese CD and UC patients compared with normal weight patients (Table 4). No overweight/obese UC patient required intestinal surgery.

We further observed, with a survival analysis based on Cox models and hazard ratios, a significant association between obesity and development of any EIM and more specifically arthritis in CD. In contrast, but not significantly, the risk of EIM and surgery is reduced in obese UC patients (Supplemental Digital Table 3, http://links.lww.com/PG9/A79).

### Quality of Life

No differences regarding QoL were observed between the BMI groups when analyzing patients with CD (Supplemental Digital Table 4, http://links.lww.com/PG9/A79). When analyzing UC patients, we found that the Kidcope-assessed escape-oriented strategy score was significantly decreased (*P* = 0.002) in overweight/obese patients (median 7, IQR 5–8) compared with UC patients with a normal weight (median 10, IQR 8–13) (Supplemental Digital Table 5, http://links.lww.com/PG9/A79). This finding indicates stronger strategies of adaptation in the overweight/obese UC group. KIDSCREEN, IMPACT III (emotional functioning, IBD symptoms, social functioning), Depressionsinventar für Kinder und Jugendliche, Kidcope control-oriented strategy, and The University of California at Los Angeles Post-traumatic Stress Disorder Reaction Index scores did not reveal differences when comparing overweight/obese UC patients with patients having normal BMI.

## DISCUSSION

Our study contains several messages of clinical relevance. First, the prevalence of overweight and obesity in the Swiss pediatric IBD cohort was 12.8%. Second, significant correlation was found between clinical activity and obesity in UC patients however with low PUCAI score corresponding to clinical remission in both obese and normal weight patients. No significant association was found between clinical or biological disease activity and being overweight and obese in CD patients. Third, overweight and obese pediatric CD patients suffered more frequently from perianal abscesses and surgery for this purpose when compared with normal weight CD patients. Fourth, in a survival analysis, obesity was positively associated with the occurrence of EIM as arthritis in CD patients. No significant association between obesity and disease complications or surgery in UC was found. And fifth, both pediatric CD and UC patients with overweight/obesity underwent similarly IBD-related hospitalization in the last 12 months when compared with normal weight patients.

Contrary to historical data suggesting the link between IBD and malnutrition, obesity tends to become more frequent in this specific population. Despite the growing knowledge about the disease, impact of obesity on adult and pediatric IBD is still poorly documented and controversial. We investigated the impact of obesity on pediatric IBD disease activity and outcome in a large Swiss cohort. Prevalence of obesity varies according to the target population and the different regions of the world. Despite a global increase in childhood obesity over the past decades, several countries, including Switzerland, have recently reported stabilizing trends (3). Prevalence of overweight and obesity in the Swiss pediatric population between 2017 and 2018 was 15.9% (3). In North American multicenter cohorts conducted between 2000 and 2002, prevalence of overweight and obesity among children with CD and UC was 9%–10% and 20%–34%, respectively (23). In our population, prevalence of 12.8% of children with overweight and obesity coincides better with the adult data of the study conducted by Greuter et al (24), where prevalence of obesity was 11%. Application of WHO Growth reference, in contrast to the definitions of the Centers for Disease Control and Prevention used in the previous studies, as well as the differences in obesity prevalence between the European and North American populations, probably explain these variations. It should be noted that a minority of pediatric patients were underweight (4%), thus confirming the hypothesis of several studies that suspicion of IBD diagnosis should be maintained despite the existence of overweight or obesity (4,23). Finally, overweight and obesity are common in pediatric IBD with a prevalence that correlates directly with data from the general pediatric population.

Although it has been shown that obesity is associated with higher disease activity and worse disease progression in rheumatoid arthritis and psoriasis, longitudinal studies show variable effect of obesity on IBD disease activity (6,9,25). We observed no significant difference concerning disease behavior, extent or biological disease activity between overweight/obese and normal weight patients. These findings coincide with two other pediatric studies stating no impact of obesity on disease activity or disease outcome in IBD patients (26,27). Obesity is considered to represent a perpetual proinflammatory state, as such, one would expect worse outcomes after several years of disease evolution. Greuter et al (24) described a negative association between obesity and clinical remission in IBD patients. In contrast to these findings, a study conducted by Flores et al (9) revealed a lower risk of IBD-related surgery, hospitalization, and initiation of anti-TNF therapy in patients who were overweight or obese compared with patients with normal weight. In our study, there were no differences with regards to disease severity corresponding to the early use of anti-TNF drugs and need for surgery in obese patients rather than in normal weight patients. Obesity might affect treatment response to biologics but studies of this data are limited.

We found significantly better QoL in overweight/obese compared with normal weight UC patients. This association of less complications, better well-being and increased weight coincide with recent studies conducted in the general population and in adult IBD patients, which have demonstrated that overweight body status may also be associated with better clinical condition and lower mortality compared with normal weight population (6,28). Despite a higher rate of perianal abscesses and related surgery, a disease course complicated by arthritis in CD patients and the hypothesis of a decreased risk of complications or surgery in UC, similar hospitalization rate was found in both CD and UC patients within the last 12 months.

Our study has several strengths and also some limitations. We report data from a well-characterized national cohort with prospective follow up that covers the majority of pediatric IBD patients in Switzerland. In contrast to many studies that have focused entirely on the relationship between obesity and disease outcomes, we report also on pediatric IBD-specific QoL. A first limitation is the limited sample size of the pediatric IBD population that was diagnosed with overweight and obesity. Second, the median follow-up time is limited in the pediatric IBD cohort. Third, due to the nature of the SIBDCS data collection, it was not possible to assess efficacy of anti-TNF treatment in obese patients compared with normal weight individuals. This type of analysis would be of particular interest given previous findings of rapid anti-TNF clearance and low trough levels in obese patients. Fourth, the methodology of the SIBDCS collecting for clinical data only at the time of inclusion and not at diagnosis, there is uncertainty about causality. In particular, we noted more obese CD treated with steroids than underweight patients. However, we cannot affirm that obese patients were not obese before steroid treatment or on the contrary that treatment favored the evolution toward an increase of the BMI.

In summary, obesity was not associated with increased disease activity, severity, or need of IBD-related hospitalization in pediatric IBD patient when compared with normal weight patient. In pediatric CD population, more complications such as development of perianal abscesses were significantly associated with obesity.

## ACKNOWLEDGMENTS

All authors approved the final version of the article.

The authors would like to thank all patients and members of the Swiss IBD cohort study group for their contribution to this work.

## Supplementary Material



## Appendix

Members of the SIBDCS study group: Karim Abdelrahman, Gentiana Ademi, Patrick Aepli, Amman Thomas, Claudia Anderegg, Anca-Teodora Antonino, Eva Archanioti, Eviano Arrigoni, Diana Bakker de Jong, Bruno Balsiger, Polat Bastürk, Peter Bauerfeind, Andrea Becocci, Dominique Belli, José M. Bengoa, Luc Biedermann, Janek Binek, Mirjam Blattmann, Stephan Boehm, Tujana Boldanova, Jan Borovicka, Christian P. Braegger, Stephan Brand, Lukas Brügger, Simon Brunner, Patrick Bühr, Bernard Burnand, Sabine Burk, Emanuel Burri, Sophie Buyse, Dahlia-Thao Cao, Ove Carstens, Dominique H. Criblez, Sophie Cunningham, Fabrizia D’Angelo, Philippe de Saussure, Lukas Degen, Joakim Delarive, Christopher Doerig, Barbara Dora, Susan Drerup, Mara Egger, Ali El-Wafa, Matthias Engelmann, Christian Felley, Markus Fliegner, Nicolas Fournier, Montserrat Fraga, Yannick Franc, Pascal Frei, Remus Frei, Michael Fried, Florian Froehlich, Raoul Ivano Furlano, Luca Garzoni, Martin Geyer, Laurent Girard, Marc Girardin, Delphine Golay, Ignaz Good, Ulrike Graf Bigler, Beat Gysi, Johannes Haarer, Marcel Halama, Janine Haldemann, Pius Heer, Benjamin Heimgartner, Beat Helbling, Peter Hengstler, Denise Herzog, Cyrill Hess, Klaas Heyland, Thomas Hinterleitner, Claudia Hirschi, Petr Hruz, Pascal Juillerat, Carolina Khalid-de Bakker, Stephan Kayser, Céline Keller, Christina Knellwolf, Christoph Knoblauch, Henrik Köhler, Rebekka Koller, Claudia Krieger, Patrizia Künzler, Rachel Kusche, Frank Serge Lehmann, Andrew Macpherson, Michel H. Maillard, Michael Manz, Astrid Marot, Rémy Meier, Christa Meyenberger, Pamela Meyer, Pierre Michetti, Benjamin Misselwitz, Patrick Mosler, Christian Mottet, Christoph Müller, Beat Müllhaupt, Leilla Musso, Michaela Neagu, Cristina Nichita, Jan Niess, Andreas Nydegger, Nicole Obialo, Diana Ollo, Cassandra Oropesa, Ulrich Peter, Daniel Peternac, Laetitia Marie Petit, Valérie Pittet, Daniel Pohl, Marc Porzner, Claudia Preissler, Nadia Raschle, Ronald Rentsch, Alexandre Restellini, Sophie Restellini, Jean-Pierre Richterich, Frederic Ris, Branislav Risti, Marc Alain Ritz, Gerhard Rogler, Nina Röhrich, Jean-Benoît Rossel, Vanessa Rueger, Monica Rusticeanu, Markus Sagmeister, Gaby Saner, Bernhard Sauter, Mikael Sawatzki, Michael Scharl, Martin Schelling, Susanne Schibli, Hugo Schlauri, Dominique Schluckebier, Daniela Schmid, Sybille Schmid, Jean-François Schnegg, Alain Schoepfer, Vivianne Seematter, Frank Seibold, Mariam Seirafi, Gian-Marco Semadeni, Arne Senning, Christiane Sokollik, Joachim Sommer, Johannes Spalinger, Holger Spangenberger, Philippe Stadler, Peter Staub, Dominic Staudenmann, Volker Stenz, Michael Steuerwald, Alex Straumann, Andreas Stulz, Michael Sulz, Aurora Tatu, Michela Tempia-Caliera, Joël Thorens, Kaspar Truninger, Radu Tutuian, Patrick Urfer, Stephan Vavricka, Francesco Viani, Jürg Vögtlin, Roland Von Känel, Dominique Vouillamoz, Rachel Vulliamy, Paul Wiesel, Reiner Wiest, Stefanie Wöhrle, Samuel Zamora, Silvan Zander, Jonas Zeitz, Dorothee Zimmermann.
