# Supplementary material for: Impact of Overweight and Obesity on Disease Outcome in the Pediatric Swiss Inflammatory Bowel Disease Cohort
Source: JPGN Rep. 2022 Mar 31;3(2):e193. doi: 10.1097/PG9.0000000000000193 (PMC10158416; doi:10.1097/PG9.0000000000000193)
Supplement: Supplementary file 1 [file pg9-3-e193-s001.pdf]

Supplementary Table 1: Baseline characteristics of CD patients.

BMI category at enrolment	underweight	normal	overweight	obese	p value
Number of patients	9 (6.2%)	120 (82.2%)	10 (6.8%)	7 (4.8%)	
Gender<br>Male<br>Female	 5 (55.6%)<br>4 (44.4%)	 62 (51.7%)<br>58 (48.3%)	 7 (70.0%)<br>3 (30.0%)	 6 (85.7%)<br>1 (14.3%)	 0.255
Age at diagnosis (yrs) (median, IQR, range)	13.2, 9.8 – 13.7,<br>2.7 – 14.6	11.5, 8.8 – 13.0,<br>0.5 – 16.0	10.3, 6.9 – 12.2,<br>3.2 – 12.9	8.5, 4.4 – 10.1,<br>3.2 – 10.3	0.034
Age at enrolment (yrs) (median, IQR, range)	14.1, 11.3 – 14.5,<br>7.2 – 15.0	12.5, 10.5 – 14.2,<br>1.3 – 17.1	13.1, 10.5 – 13.5,<br>4.0 – 13.9	10.2, 5.0 – 11.3,<br>3.8 – 13.0	0.110
Disease duration (yrs) at enrolment (median, IQR, range)	1.2, 0.6 – 1.7,<br>0.3 – 4.9	0.9, 0.5 – 1.7,<br>0.2 – 8.7	1.1, 0.6 – 2.8,<br>0.5 – 9.7	1.0, 0.6 – 3.0,<br>0.5 – 5.3	0.528
Follow-up time (yrs) (median, IQR, range)	1.9, 1.1 – 2.0,<br>0 – 5.5	2.5, 0.9 – 4.2,<br>0 – 9.5	3.3, 2.1 – 5.6,<br>1.8 – 9.7	2.0, 0 – 2.4,<br>0 – 3.4	0.110
Current treatments<br>5-ASA oral<br>5-ASA topical<br>Prednisone<br>Budesonide<br>Thiopurines<br>Methotrexate<br>CNI<br>Anti-TNF<br>Vedolizumab<br>ustekinumab	 1 (11.1%)<br>0 (0%)<br>1 (11.1%)<br>0 (0%)<br>3 (33.3%)<br>0 (0%)<br>0 (0%)<br>4 (44.4%)<br>0 (0%)<br>0 (0%)	 5 (4.2%)<br>0 (0%)<br>23 (19.2%)<br>1 (0.8%)<br>39 (32.5%)<br>20 (16.7%)<br>0 (0%)<br>72 (60.0%)<br>3 (2.5%)<br>3 (2.5%)	 2 (20.0%)<br>0 (0%)<br>2 (20.0%)<br>0 (0%)<br>3 (30.0%)<br>2 (20.0%)<br>0 (0%)<br>7 (70.0%)<br>1 (10.0%)<br>1 (10.0%)	 0 (0%)<br>0 (0%)<br>0 (0%)<br>1 (14.3%)<br>4 (57.1%)<br>0 (0%)<br>0 (0%)<br>2 (28.6%)<br>1 (14.3%)<br>0 (0%)	 0.141<br>-<br>0.792<br>0.110<br>0.601<br>0.486<br>-<br>0.276<br>0.168<br>0.548
Ever treated with…<br>5-ASA oral<br>5-ASA topical<br>Prednisone<br>Budesonide<br>Thiopurines<br>Methotrexate<br>CNI<br>Anti-TNF<br>Vedolizumab<br>Ustekinumab	 5 (55.6%)<br>0 (0%)<br>2 (22.2%)<br>1 (11.1%)<br>8 (88.9%)<br>2 (22.2%)<br>0 (0%)<br>4 (44.4%)<br>0 (0%)<br>0 (0%)	 33 (27.5%)<br>12 (10.0%)<br>85 (70.8%)<br>23 (19.2%)<br>102 (85.0%)<br>36 (30.0%)<br>2 (1.7%)<br>84 (70.0%)<br>4 (3.3%)<br>3 (2.5%)	 5 (50.0%)<br>2 (20.0%)<br>7 (70.0%)<br>3 (30.0%)<br>7 (70.0%)<br>5 (50.0%)<br>0 (0%)<br>9 (90.0%)<br>2 (20.0%)<br>1 (10.0%)	 2 (28.6%)<br>0 (0%)<br>6 (85.7%)<br>1 (14.3%)<br>7 (100.0%)<br>0 (0%)<br>0 (0%)<br>3 (42.9%)<br>1 (14.3%)<br>0 (0%)	 0.172<br>0.500<br>0.021<br>0.781<br>0.431<br>0.172<br>1.000<br>0.073<br>0.060<br>0.548
History of surgery<br>No<br>Yes	 7 (77.8%)<br>2 (22.2%)	 105 (87.5%)<br>15 (12.5%)	 6 (60.0%)<br>4 (40.0%)	 5 (71.4%)<br>2 (28.6%)	 0.050
Smoking status at latest follow-up<br>Non-Smoker<br>Smoker<br>Unknown	8 (88.9%)<br>1 (11.1%)<br>0 (0%)	111 (92.5%)<br>8 (6.7%)<br>1 (0.8%)	10 (100%)<br>0 (0%)<br>0 (0%)	7 (100%)<br>0 (0%)<br>0 (0%)	0.762

Abbreviations: 5-ASA, aminosalicylates; BMI, body mass index; CD, Crohn's disease; CNI, calcineurin inhibitors; IQR, interquartile range; TNF, tumor necrosis factor

Supplementary Table 2: Baseline characteristics of UC patients.

BMI category at enrolment	underweight	normal	overweight	obese	p value
Number of patients	4 (2.2%)	152 (84.0%)	12 (6.6%)	13 (7.2%)	
Gender					
Male	3 (75.0%)	72 (47.4%)	6 (50.0%)	10 (76.9%)	0.156
Female	1 (25.0%)	80 (52.6%)	6 (50.0%)	3 (23.1%)	
Age at diagnosis (yrs) (median, IQR, range)	5.9, 2.5 – 15.4, 2.5 – 15.4	11.2, 8.0 – 13.8, 0.8 – 16.3	9.1, 5.1 – 11.7, 3.1 – 17.2	10.5, 6.8 – 11.4, 3.2 – 14.9	0.356
Age at enrolment (yrs) (median, IQR, range)	9.6, 5.2 – 14.3, 3.3 – 16.5	13.0, 9.4 – 14.9, 1.5 – 16.7	11.4, 7.6 – 14.5, 3.9 – 17.6	11.8, 8.4 – 13.1, 3.9 – 15.3	0.371
Disease duration (yrs) at enrolment (median, IQR, range)	1.1, 0.8 – 1.1, 0.8 – 1.1	0.8, 0.5 – 1.8, 0.2 – 9.9	1.8, 0.7 – 3.0, 0.4 – 5.8	0.6, 0.6 – 1.4, 0.3 – 4.2	0.468
Follow-up time (yrs) (median, IQR, range)	4.7, 3.2 – 5.2, 2.1 – 5.2	1.8, 0.2 – 3.5, 0 – 9.4	1.6, 0 – 2.9, 0 – 4.9	3.2, 2.9 – 4.7, 0 – 8.3	0.009
Current treatments					
5-ASA oral	1 (25.0%)	101 (66.4%)	7 (58.3%)	9 (69.2%)	0.348
5-ASA topical	1 (25.0%)	24 (15.8%)	4 (33.3%)	2 (15.4%)	0.365
Prednisone	0 (0%)	23 (15.1%)	2 (16.7%)	2 (15.4%)	1.000
Budesonide	0 (0%)	2 (1.3%)	0 (0%)	0 (0%)	1.000
Thiopurines	1 (25.0%)	52 (34.2%)	5 (41.7%)	4 (30.8%)	0.956
Methotrexate	0 (0%)	15 (9.9%)	0 (0%)	1 (7.7%)	0.894
CNI	0 (0%)	1 (0.7%)	0 (0%)	0 (0%)	1.000
Anti-TNF	2 (50.0%)	37 (24.3%)	0 (0%)	5 (38.5%)	0.044
Vedolizumab	0 (0%)	4 (2.6%)	0 (0%)	1 (7.7%)	0.587
Ustekinumab	0 (0%)	0 (0%)	0 (0%)	0 (0%)	-
Ever treated with…					
5-ASA oral	4 (100%)	137 (90.1%)	11 (91.7%)	13 (100%)	0.813
5-ASA topical	2 (50.0%)	68 (44.7%)	7 (58.3%)	7 (53.8%)	0.766
Prednisone	3 (75.0%)	106 (69.7%)	4 (33.3%)	10 (76.9%)	0.063
Budesonide	1 (25.0%)	19 (12.5%)	1 (8.3%)	5 (38.5%)	0.058
Thiopurines	3 (75.0%)	86 (56.6%)	6 (50.0%)	9 (69.2%)	0.711
Methotrexate	1 (25.0%)	23 (15.1%)	1 (8.3%)	2 (15.4%)	0.820
CNI	1 (25.0%)	7 (4.6%)	0 (0%)	0 (0%)	0.330
Anti-TNF	3 (75.0%)	48 (31.6%)	1 (8.3%)	5 (38.5%)	0.077
Vedolizumab	1 (25.0%)	6 (3.9%)	0 (0%)	1 (7.7%)	0.186
Ustekinumab	0 (0%)	0 (0%)	0 (0%)	0 (0%)	-
History of surgery					
No	3 (75.0%)	144 (94.7%)	11 (91.7%)	13 (100%)	0.204
Yes	1 (25.0%)	8 (5.3%)	1 (8.3%)	0 (0%)	
Smoking status at latest follow-up					
Non-Smoker	4 (100%)	141 (92.8%)	11 (91.7%)	13 (100%)	0.691
Smoker	0 (0%)	7 (4.6%)	0 (0%)	0 (0%)	
Unknown	0 (0%)	4 (2.6%)	1 (8.3%)	0 (0%)	

Abbreviations: 5-ASA, aminosalicylates; BMI, body mass index; CNI, calcineurin inhibitors; IQR, interquartile range; TNF, tumor necrosis factor; UC, ulcerative colitis

Supplementary Table 3: Survival analysis, stratified by diagnosis. Adjustment is done for age, sex, steroid intake, and initial disease location.

	CD, crude HR (95% CI; p)	CD, adjusted HR (95% CI; p)	UC, crude HR (95% CI; p)	UC, adjusted HR (95% CI; p)
Extraintestinal manifestations				
Arthritis	1.160 (1.021 – 1.319; p=0.023)	1.187 (1.044 – 1.350; p=0.009)	0.933 (0.796 – 1.093; p=0.389)	0.941 (0.800 – 1.107; p=0.463)
Oral ulcers	1.077 (0.946 – 1.225; p=0.262)	1.110 (0.965 – 1.277; p=0.145)	0.883 (0.751 – 1.038; p=0.130)	0.868 (0.728 – 1.035; 0.114)
Any	1.093 (0.999 – 1.195; p=0.053)	1.111 (1.013 – 1.218; p=0.026)	0.905 (0.809 – 1.013; p=0.081)	0.904 (0.803 – 1.017; p=0.092)
Any surgery	1.049 (0.838 – 1.313; p=0.675)	1.063 (0.858 – 1.317; p=0.575)	0.918 (0.752 – 1.121; p=0.401)	0.903 (0.730 – 1.117; p=0.348)
Any fistula	1.014 (0.893 – 1.152; p=0.828)	1.009 (0.882 – 1.154; p=0.897)		

Abbreviations: CD, Crohn’s disease; CI, confidence interval; HR, hazard ratio; UC, ulcerative colitis

Supplementary Table 4: QoL in CD patients, assessed at latest follow-up.

BMI category at enrolment	Normal BMI (n=120)	Overweight/obese (n=17)	P value
KIDSCREEN QUESTIONNAIRE TOTAL (median, IQR, range)	115, 106 – 123, 72 – 134	117, 103 – 126, 72 – 134	0.476
KIDSCREEN PHYSICAL ACTIVITY (median, IQR, range)	20, 16.5 – 21, 7 – 25	19, 14 – 22, 11 – 25	0.906
KIDSCREEN MOOD (median, IQR, range)	30, 28 – 33, 18 – 35	30.5, 26 – 35, 18 – 35	0.654
KIDSCREEN FAMILY LIFE (median, IQR, range)	32, 29 – 33, 16 – 35	32.5, 29 – 35, 20 – 35	0.387
KIDSCREEN FRIENDS (median, IQR, range)	18, 16 – 19, 6 – 20	18, 16 – 19, 11 – 20	0.626
KIDSCREEN SCHOOL (median, IQR, range)	16, 15 – 18, 8 – 20	18, 16 – 19, 8 – 20	0.108
DIKJ QUESTIONNAIRE STEINMEIER POLSTER 1989 (median, IQR, range)	7, 4 – 11, 0 – 23	8.5, 6 – 11.4, 3 – 29	0.292
KIDCOPE QUESTIONNAIRE (ADAPTATION STRATEGY) SPIRITO 1988			
Control-oriented strategies (median, IQR, range)	9, 7 – 11,1 – 18	9, 6 – 10, 3 – 12	0.371
Escape-oriented strategies (median, IQR, range)	9, 7 – 11.5, 2 – 21	11, 9 – 12, 3 – 20	0.227
UCLA PTSD INDEX STEINBERN 2004, STRESS SYMPTOM QUESTIONNAIRE: existence of a trauma history			
No	50 (56.8%)	11 (78.6%)	0.151
Yes	38 (43.2%)	3 (21.4%)	
Missing	*32*	*3*	
UCLA PTSD INDEX STEINBERN 2004, STRESS SYMPTOM QUESTIONNAIRE			
median, IQR, range	14, 10 – 25, 0 – 41	13, 8.5 – 29.5, 7 – 43	0.922

Abbreviations: BMI, body mass index; CD, Crohn’s disease; IQR, interquartile range; QoL quality of life

Supplementary Table 5: QoL in UC patients, assessed at latest follow-up.

BMI category at enrolment	Normal BMI (n=152)	Overweight/obese (n=25)	P value
KIDSCREEN QUESTIONNAIRE TOTAL (median, IQR, range)	116, 106 – 124, 71 – 134	122, 106 – 127, 80 – 133	0.378
KIDSCREEN PHYSICAL ACTIVITY (median, IQR, range)	19, 16.5 – 22, 9 – 25	19, 15 – 21, 9 – 24	0.571
KIDSCREEN MOOD (median, IQR, range)	31, 28 – 33.4, 17 – 35	31, 30 – 34, 22 – 35	0.541
KIDSCREEN FAMILY LIFE (median, IQR, range)	32, 28.5 – 34, 14 – 35	33, 31 – 35, 20 – 35	0.325
KIDSCREEN FRIENDS (median, IQR, range)	18, 16 – 19, 4 – 20	19, 17 – 20, 9 – 20	0.203
KIDSCREEN SCHOOL (median, IQR, range)	17, 14 – 19, 8 – 20	17, 16 – 19, 9 – 20	0.327
DIKJ QUESTIONNAIRE STEINMEIER POLSTER 1989 (median, IQR, range)	6, 3 – 12, 0 – 33	6, 1 – 10.4, 0 – 30	0.385
KIDCOPE QUESTIONNAIRE (ADAPTATION STRATEGY) SPIRITO 1988			
Control-oriented strategies (median, IQR, range)	10, 7 – 12, 0 – 16	9, 6 – 12, 4 – 16	0.419
Escape-oriented strategies (median, IQR, range)	10, 8 – 13, 3 – 19	7, 5 – 8, 1 – 17	0.002
UCLA PTSD INDEX STEINBERN 2004, STRESS SYMPTOM QUESTIONNAIRE: existence of a trauma history			
No	61 (62.2%)	9 (64.3%)	1.000
Yes	37 (37.8%)	5 (35.7%)	
Missing	*54*	*11*	
UCLA PTSD INDEX STEINBERN 2004, STRESS SYMPTOM QUESTIONNAIRE (median, IQR, range)	18, 11 – 27, 0 – 51	22.9, 8.9 – 30.5, 0 – 33	0.873

Abbreviations: BMI, body mass index; IQR, interquartile range; QoL, quality of life; UC, ulcerative colitis
